# Unloading of the Left Ventricle With More Delayed Reperfusion May Reduce Reperfusion Injury

**DOI:** 10.7759/cureus.52831

**Published:** 2024-01-23

**Authors:** Sara Hazaveh, Haroon Faraz

**Affiliations:** 1 Internal Medicine, Hackensack University Medical Center, Hackensack, USA; 2 Interventional Cardiology, Hackensack University Medical Center, Hackensack, USA

**Keywords:** cardiac catherization, left ventricular unloading, door to balloon time, reperfusion injury, acute st-elevation myocardial infarction

## Abstract

Early reperfusion therapy is crucial and the standard of care for the management of acute ST-elevation myocardial infarction (STEMI). We report a case of STEMI with unloading followed by more delayed reperfusion, which challenges current clinical practice. It also highlights the importance of more translational research to better understand STEMI on a mechanistic level including the crucial role of mitochondria and anaerobic respiration during vessel occlusion and ischemia. This can also help in preventing post-myocardial infarction complications such as reperfusion injury, which leads to the development of heart failure.

## Introduction

Successful management of acute myocardial infarction (AMI) emphasizes early reperfusion therapy for decades and heart failure is a major complication of AMI [1]. Further reduction in door-to-balloon (DTB) times has not improved post-AMI heart failure and mortality [2]. Preclinical studies and the DTU-STEMI clinical trial (Door-To-Unload in ST-Segment-Elevation Myocardial Infarction Pilot Trial) have suggested that mechanically unloading the left ventricle (LV) prior to reperfusion for 30 minutes can reduce infarct size and myocardial injury in STEMI without cardiogenic shock by reducing reperfusion injury [3-7]. Here we present a case of multivessel STEMI complicated with cardiogenic shock that had significant myocardial recovery by early unloading of LV followed by delayed reperfusion, which challenges current practice and LV unloading with delayed reperfusion studies.

## Case presentation

**Figure 1:**
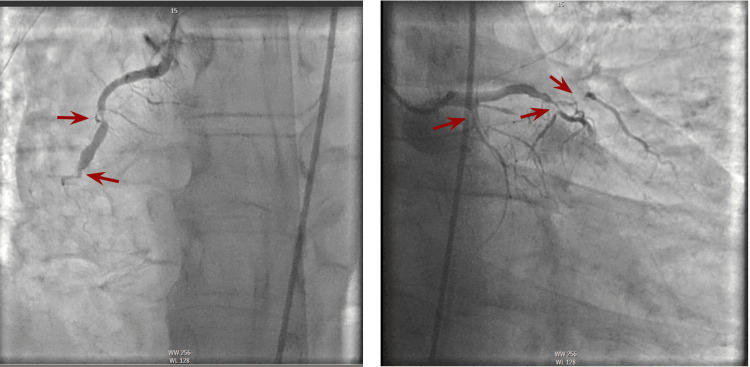
Cardiac catheterization prior to establishing reperfusion. Occluded right coronary artery (RCA, left image) and occluded left anterior ascending artery (LAD, right), red arrows representing vessel stenosis. He underwent successful stenting and reperfusion of occluded RCA at 16:17 PM and stending and reperfusion of occluded LAD at 16:52 PM.

A 49-year-old male with a 30-pack-a-year smoking history started to experience typical AMI symptoms while driving at 10:00 AM. Emergency medical services (EMS) were called and the patient was taken to a local community hospital. He arrived there at 11:38 AM. An electrocardiogram was done en route showing anterolateral wall STEMI. The STEMI code was activated on arrival at the hospital. The initial lab results were notable for lactate 2.5, troponin-I 4.60 ng/mL (normal <0.4 ng/mL). At 11:52 AM he was emergently taken to cardiac catheterization laboratory; he was then found to be hypotensive on transfer to the catheterization table. Norepinephrine and vasopressin were started for hemodynamic support and he required emergent intubation for airway protection. Left heart catheterization (LHC) at approximately 12:00 PM revealed an LV end-diastolic pressure (LVEDP) of 38 and LV ejection fraction (LVEF) of <20% with an occluded right coronary artery (RCA) and occluded left anterior descending artery (LAD) (Figure 1).

**Figure 2:**
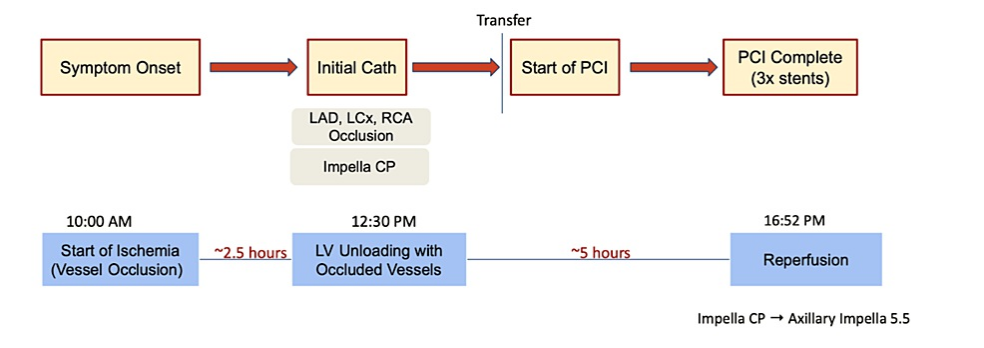
Timeline of ischemia onset-unloading and reperfusion. Cath: catheterization; PCI: percutaneous coronary intervention; LAD: left anterior descending artery; LCx: left circumflex artery; RCA: right coronary artery; LV: left ventricle Impella CP: Impella Cardiac Power (Abiomed, Inc., Danvers, Massachusetts, United States)

An Impella Cardiac Power (CP) device (Abiomed, Inc., Danvers, Massachusetts, United States) was placed for myocardial protection and LV unloading, and the patient was emergently transferred to our tertiary care center for advanced interventions. The patient was taken to our catheterization laboratory where he underwent successful stenting and reperfusion of the occluded RCA at 16:17 PM and of the occluded LAD at 16:52 PM. This patient had 2.5 hours from the time of ischemia onset to unloading via the Impella device and five hours from unloading to the establishment of reperfusion via catheterization in his case of AMI. More specifically, the time of ischemia onset is when a patient starts to experience his symptoms, and the time of reperfusion is when he has stent placement to relieve the coronary stenosis. This timeline is better represented in Figure 2. Post complete revascularization, the Impella CP was upgraded to Impella axillary 5.5 due to mild hematuria and continued pressor requirements. He was subsequently extubated a day after catheterization and the Impella device was removed a few hours after that. The percutaneous coronary intervention (PCI) LVEF was 15% with severe left ventricular hypokinesis. One week later, the echocardiogram showed improvements in LVEF to 35-40%.

## Discussion

AMI is the leading cause of death and heart failure in the United States. The incidence of post-AMI heart failure remains high despite all strict efforts in resource allocation to achieve DTB times <90 minutes. Every 5% increase in myocardial infarct size is associated with a 20% increase in one-year hospitalization for heart failure and one-year mortality [1,2]. New approaches for AMI management are needed as efforts to reduce total ischemic time have not reduced post-AMI complications. 

Preclinical models over the past 20 years have shown that in comparison with reperfusion alone, mechanically unloading the LV before establishing coronary reperfusion in AMI reduces infarct size by limiting reperfusion injury [5,6]. Delaying reperfusion by 30 minutes has also been shown to activate a cardioprotective signaling program that reduces ischemia-reperfusion damage and can help with myocardial recovery 30 days after AMI [7]. The DTU-STEMI trial is the pilot clinical study investigating the safety and efficacy of door-to-unloading time using the Impella CP device with a 30-minute delay prior to reperfusion and has shown that it is feasible without suggesting any safety concerns [7].

Possible explanations for the benefits of delayed perfusion include a reduction in endothelin-1 and thus calcium release or the development of microcirculatory collateral blood flow to the myocardium at risk [8,9]. On a mechanistic level, LV unloading can maintain mitochondrial morphology, function, and cardiolipin levels in the infarcted zone compared to reperfusion alone [10]. Cardiolipin is an integral part of the inner mitochondrial membrane that regulates various mitochondrial proteins such as the electron transport complexes that are crucial for anaerobic respiration. The duration that mitochondria and associated signaling mechanisms can remain functional under anaerobic conditions hasn't been explored.

## Conclusions

The presented case had an LV unloading with delayed perfusion time more than what has been used in studies suggesting that mitochondrial mechanisms under anaerobic conditions may be functional for longer periods. Additionally, reperfusion injury which contributed to post-AMI LV dysfunction was prevented by unloading and delayed reperfusion. Given the central role of mitochondria for anaerobic respiration during ischemia and reperfusion injury, more studies exploring mitochondrial integrity with unloading and delayed reperfusion are needed. Additionally, the duration of time that anaerobic respiration can be functional needs to be explored.

## References

[1] Benjamin EJ, Blaha MJ, Chiuve SE (2017). Heart disease and stroke statistics-2017 update: a report from the American Heart Association. Circulation.

[2] Menees DS, Peterson ED, Wang Y, Curtis JP, Messenger JC, Rumsfeld JS, Gurm HS (2013). Door-to-balloon time and mortality among patients undergoing primary PCI. N Engl J Med.

[3] Stone GW, Selker HP, Thiele H (2016). Relationship between infarct size and outcomes following primary PCI: patient-level analysis from 10 randomized trials. J Am Coll Cardiol.

[4] Ezekowitz JA, Kaul P, Bakal JA, Armstrong PW, Welsh RC, McAlister FA (2009). Declining in-hospital mortality and increasing heart failure incidence in elderly patients with first myocardial infarction. J Am Coll Cardiol.

[5] Kapur NK, Reyelt L, Swain L (2019). Mechanical left ventricular unloading to reduce infarct size during acute myocardial infarction: insight from preclinical and clinical studies. J Cardiovasc Transl Res.

[6] Khalid N, Shlofmitz E, Waksman R (2019). Letter by khalid et al regarding article, “unloading the left ventricle before reperfusion in patients with anterior ST-segment-elevation myocardial infarction: a pilot study using the Impella CP”. Circulation.

[7] Parikh MJ, Schuleri KH, Chakrabarti AK, O'Neill WW, Kapur NK, Wohns DH (2021). Door-to-unload: left ventricular unloading before reperfusion in ST-elevation myocardial infarction. Future Cardiol.

[8] LeDoux JF, Tamareille S, Felli PR, Amirian J, Smalling RW (2008). Left ventricular unloading with intra-aortic counter pulsation prior to reperfusion reduces myocardial release of endothelin-1 and decreases infarction size in a porcine ischemia-reperfusion model. Catheter Cardiovasc Interv.

[9] Zografos TA, Katritsis DG (2016). Cyclosporine before PCI in acute myocardial infarction. N Engl J Med.

[10] Curran J, Burkhoff D, Kloner RA (2019). Beyond reperfusion: acute ventricular unloading and cardioprotection during myocardial infarction. J Cardiovasc Transl Res.

